# Outcome Measures of Chinese Herbal Medicine for Hypertension: An Overview of Systematic Reviews

**DOI:** 10.1155/2012/697237

**Published:** 2012-12-24

**Authors:** Jie Wang, Xingjiang Xiong

**Affiliations:** Department of Cardiology, Guang'anmen Hospital, China Academy of Chinese Medical Sciences, Beijing 100053, China

## Abstract

*Objective.* The aim of this overview was to summarize the outcome measures of Chinese herbal medicine (CHM) for the treatment of hypertension based on available systematic reviews (SRs), so as to evaluate the potential benefits and advantages of CHM on hypertension. *Methods.* Literature searches were conducted in the Cochrane Database of Systematic Reviews, MEDLINE, and 4 databases in Chinese. SRs of CHM for hypertension were included. Two independent reviewers (J. Wang and X. J. Xiong) extracted the data. *Results.* 10 SRs were included. 2 SRs had primary endpoints, while others focused on secondary endpoints to evaluate CHM for hypertension such as blood pressure (BP) and Traditional Chinese Medicine (TCM) syndrome. 6 SRs have reported the adverse effects, whereas the other 4 SRs have not mentioned it at all. Many CHM appeared to have significant effect on improving BP, TCM syndrome, and so on. However, most SRs failed to make a definite conclusion for the effectiveness of CHM for hypertension due to poor evidence. *Conclusion.* Primary endpoints have not been widely used currently. The benefits of CHM for hypertension need to be confirmed in the future with randomized controlled trials (RCTs) of more persuasive primary endpoints and high-quality SRs.

## 1. Introduction

Hypertension is one of the most common and important health problems affecting millions of people throughout the world and about 20% of the adult population in many countries [1]. It could lead to severe complications, such as hypertensive cardiovascular disease, hypertensive renal disease, and atherosclerotic complications including stroke, coronary heart disease, renal insufficiency, and heart failure [2]. However, hypertension in most individuals remains untreated or uncontrolled [3]. Effective treatment of hypertension is limited by availability, cost, and adverse effects of antihypertensive medications. Some hypertension-related symptoms could not be completely relieved by conventional medicine. Hypertension is the major cause of morbidity and mortality and is the third highest risk factor for lifetime burden worldwide [4]. Therefore, some patients have turned to complementary and alternative therapies (or traditional medicine), especially Chinese medicine (CM) [5–9], hoping that such treatments might improve their symptoms. Chinese medicine (CM) has a history for more than 2500 years with unique theory of diagnosis and treatment [10–15]. In recent years, with the popularity and prevalence of Chinese medicine (CM), there has been a growing interest in Chinese herbal medicine (CHM) for patients with hypertension both in China and the West [16–20]. Until now a number of clinical studies of CHM reported the clinical effectiveness in hypertensive patients ranging from case reports and case series to controlled observational studies and randomized clinical trials. However, the evidence needs to be reviewed systematically [21]. 

As the evidence gathering tools, systematic reviews (SRs) of randomized controlled trials (RCTs) are considered to provide the best evidence about the effectiveness of interventions [22, 23]. Physicians and policy makers need evidence from SRs for decision making and policy making. Patients and researchers also need such information to support shared decisions and to set priorities for research. Recently, an increasing number of SRs about CHM for hypertension have been reported. However, few of them have shown that CHM was definitely effective for hypertension due to the weak evidence. There is a need for combining multiple reviews into overviews to provide users with easily available information. In addition, when people making decisions about health care look for guidance from research, the outcomes reported are key, which also plays an important role in drawing a more persuasive conclusion [24]. However, there is a general lack of consensus regarding the choice of outcomes in particular clinical settings, which affect trial design, conduct, analysis, and reporting [25]. The aim of this overview was to summarize the outcome measures of CHM for treatment of hypertension based on available SRs both in English and Chinese, so as to display the current situation and evaluate the potential benefits and advantages of CHM on hypertension.

## 2. Methods

Literature searches were conducted in the Cochrane Database of Systematic Review (October, 2012), MEDLINE (2002–2012), Chinese National Knowledge Infrastructure (CNKI, 2002–2012), Chinese Biomedical Literature Database (CBM, 2002–2012), Chinese Scientific Journal Database (VIP, 2002–2012), and Wanfang Databases (2002–2012). All of those searches ended on October 10, 2012. CNKI, CBM, VIP, and Wanfang were four main databases in China. All of the databases in Chinese were searched to retrieve the maximum possible number of systematic reviews or meta-analyses of CHM for hypertension because CHMs are mainly used and researched in China. We searched papers from 2002 to 2012 for high-quality RCTs and SRs mainly focusing on the recent ten years.

**Figure 1:**
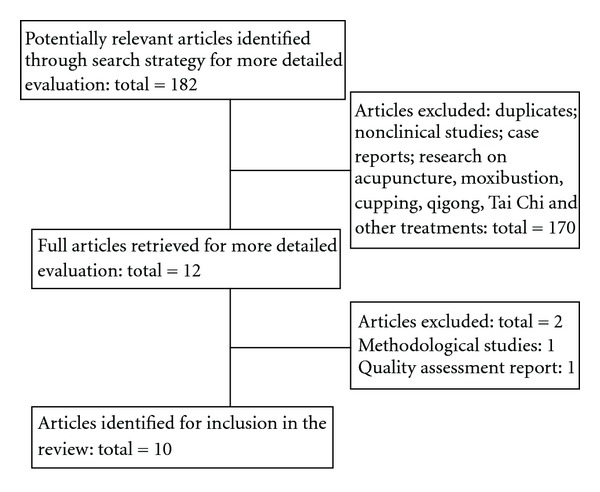
Flow-chart of SRs selection.

The strategy below was used to search The Cochrane Library and adapted appropriately for use in different electronic databases: #1 herb*; #2 medic*; #3 (#1 and #2); #4 Chinese; #5 (#3 or #4); #6 blood pressure; #7 hypertension; #8 high blood pressure; #9 (#6 or #7 or #8); #10 (#5 and #9). Two reviewers (J. Wang and X. J. Xiong) independently scanned the relevance of all references based on title and abstract of each record. If the information included a systematic review or a meta-analysis of CHM for hypertension, the full paper was obtained for further assessment. Papers were excluded when problems occurred with: repeat publication, methodological studies, quality assessment report, research on acupuncture, qigong, massage, or other treatments (Figure 1).

Outcome measures included primary endpoints and secondary endpoints. Primary endpoints include mortality, stroke, coronary heart disease, and hypertensive renal damage. Secondary endpoints mainly indicate blood pressure, the level of blood lipids, pulse pressure (PP), quality of life (QOL), and Traditional Chinese Medicine (TCM) syndrome. In addition, PRISMA (Preferred Reporting Items for Systematic reviews and Meta-Analyses) was used as an assessment tool to evaluate the quality of the included SRs [26]. As shown in the article written by Moher et al. [26], the checklist consists of 27 items in 7 key areas and a four-phase flow diagram in order to help authors improve the reporting of systematic reviews and meta-analyses. It describes the preferred way to present the title, abstract, introduction, methods, results, discussion, and funding sections in detail of systematic reviews and meta-analyses. It requires each reviewer to follow the research process and include a flow diagram providing information about the number of studies identified, included, and excluded through database searching and other sources, and reasons for excluding them such as duplicates. Information of each included SRs was imported into PRISMA statement for analysis. One author (J. Wang or X. J. Xiong) independently extracted data from each included review using predefined criteria and discussed the data with the other author to reach a consensus when there is a disagreement. 

## 3. Results

After primary search of 6 databases, 182 articles were screened out from electronic and manual searches (as shown in Figure 1), and the majority were excluded due to obvious ineligibility which including irrelevant titles and abstracts (some papers were found from more than one database). After reading the titles and abstracts, a majority of them were excluded. 170 articles were excluded because of duplicates, nonclinical studies, case reports, and research on acupuncture, moxibustion, cupping, qigong, Tai Chi, and other treatments. Then 12 full articles were retrieved for more detailed evaluation. Due to methodological study and quality assessment report, 2 out of them were excluded respectively based on the assessment tool. In the end, 10 SRs were reviewed [27–36]. All the SRs were conducted in China with 1 in English and 9 in Chinese. 9 SRs from the Chinese electronic databases were published between 2006 and 2012. Since 2011, the number of SRs increased markedly. Only 1 SR from the Cochrane Database of Systematic Review was published in 2012 [31]. 

8 SRs were concerned with essential hypertension, and the other 2 were related to elderly hypertension. We also retrieved the related clinical trials for further analysis. These clinical trials in SRs were mainly conducted in China. The methodological quality of clinical trials was assessed independently with criteria from the Cochrane Handbook for Systematic Review of Interventions, Version 5.1.0 (J. Wang and X. J. Xiong) [37]. The items included random sequence generation (selection bias), allocation concealment (selection bias), blinding of participants and personnel (performance bias), blinding of outcome assessment (detection bias), incomplete outcome data (attrition bias), selective reporting (reporting bias), and other bias. It was found out that although the original trials included all claimed “RCTs” or “quasi-RCTs”, only few of them were typical RCTs. Almost all the trials mentioned that “patients were randomized into two groups” without detailed information about randomization. So, it is hard to judge whether randomization was conducted properly and really. Most of them have not mentioned allocation concealment and double-blind. That is to say, the claimed RCTs may not be true RCTs actually. Therefore, most of the trials in the SRs were of low quality. However, only 10 RCTs were of high quality: three were concerned with replenishing spleen and kidney therapy, one was related to promoting blood circulation and removing blood stasis therapy, two were associated with clearing heat therapy such as *Bidens bipinnata L. *and *Qinre jiangya* mixture, and four were about calming the liver therapy such as *Pingjiangyin* capsule, *Pinggan jiangya* capsule, *Niuhuang jiangya* tablet, and *Tiaopingkang* tablet. Among these 10 SRs, 3 kinds of CHM were reviewed, including capsules, pellets, and herbal decoction as follows: *Niuhuang Jiangya* preparation (*n* = 1) [27]; *Tianma Gouteng *Yin (*n* = 2) [30, 31]; herbal decoction (*n* = 7) [28, 29, 32–36]. The characteristics of 10 SRs were summarized in Table 1. 

As shown Table 1, 2 SRs analyzed primary endpoints and the remaining nine SRs all focused on secondary endpoints to evaluate CHM for hypertension. This is mainly due to whether there was detailed information in the original research or not. 4 primary endpoints were analyzed in 2 SRs including essential hypertension and elderly hypertension. 1 SR about *Niuhuang Jiangya* preparation showed no effect on the mortality, stroke, coronary heart disease, and hypertensive renal damage [27]. The other 1 SR about herbal products appeared to be effective on improving hypertensive renal damage [36]. Blood pressure was the most common secondary endpoint in the SRs. All the included SRs reported blood pressure changes. Among them, 8 SRs showed improvement in blood pressure, but the other 2 SRs showed insufficient evidence [27, 31]. 5 SRs analyzed TCM syndrome changes [32–36]. There are 3 SRs that reported Triglycerides (TG) [28], pulse pressure (PP) [35], and quality of life (QOL) [36], respectively. Many CHMs appear to be effective on improving signs and symptoms, level of blood lipids, and so forth. Some SRs also reflected that CHM may be effective to prevent progression to severe complications of hypertension. However, due to poor methodological quality in the majority of included trials, most SRs could not draw confirmative conclusions on the beneficial effect of CHM for hypertension. 

Adverse effects, providing a guideline to both doctors and patients for reasonable medication, should also be regarded as an essential outcome measure in clinical trials [38, 39]. However, there is a widespread misunderstanding of CHM. Most people, especially in East Asia, think that the application of TCM has a long history, natural origination, good health care effects, efficacy of treating symptoms and root causes, and no toxic and side effects [40–42]. Even it is widely accepted that it is safe to use herbal medicines for various diseases in China. However, along with the development of pharmacology study, there are more and more reports of liver toxicity and other adverse events associated with CHM [43–45], so this paper makes the analysis on the adverse effects of CHM for hypertension. In this paper, adverse effects are ignored. 6 SRs [27, 30, 32–34, 36] have reported the adverse effects, whereas the other 4 SRs [28, 29, 31, 35] have not mentioned it at all. Only 2 trials in the 1 SR [34] had long-term data on adverse effects. Most of adverse effects of CHM were mentioned as “none obvious,” “low adverse effect” or even “no adverse effect.” The reported adverse reactions in control groups were more severe than in treatment groups. Adverse events reported in 4 SRs [27, 30, 32, 34], including headache, dizziness, cough, dry stool, and diarrhea. Thus, adverse reactions of CHM should be highlighted in systematic reviews, and the safety of CHMs needs to be monitored rigorously and reported appropriately in the future clinical trials. 

The Cochrane Collaboration is an international organization which aims to prepare and maintain rigorous systematic reviews in order to help people make well-informed decisions about health care [46]. As we know that Cochrane reviews are regarded as the highest standard of evidence with a greater methodological quality [47]. Outcome measures of the included SRs in Cochrane Database of Systematic Reviews are more credible than non-Cochrane reviews [48, 49]. They adopt primary endpoints, secondary endpoints, and safety as outcome measures. Unfortunately, in our paper, only one SR about *Tianma Gouteng* Yin for essential hypertension was retrieved from Cochrane Library [31]. The authors of the SR identified no study which met the inclusion criteria for review. As no trials could be identified for the review, no conclusions can be made about the role of *Tianma Gouteng* Yin in the treatment of essential hypertension. When referring to non-Cochrane reviews, primary endpoints and adverse effects are seldom taken as outcome measures in most SRs. 

In addition, it was found out that most of the included SRs were generally of low quality according to PRISMA statement. Review methods were not fully reported in most SRs. The characteristics of included clinical trials were not described with detailed information in 5 SRs [29, 30, 32–34]. No flow-chart of information through the different phases of a systematic review was provided. Sensitivity analysis, subgroup analysis, and potential publication bias were not analyzed sufficiently in the reviews. Convincing outcome measures were lacked in most SRs. 

## 4. Discussion

In our overview, the primary endpoints and secondary endpoints are all used to evaluate the efficacy of CHM for hypertension. It is widely known that the primary goal of essential hypertension treatment is to reduce mortality, or prevent progression to severe complications such as stroke, coronary heart disease, heart failure, and hypertensive renal damage. However, there is a lack of data on the final indicator at endpoint. Most of the included SRs have not reported the mortality rate or the incidence of complications. The primary endpoints are seldom used due to the difficulty of clinical implementation, limitations of the research funding and other reasons. On the contrary, secondary endpoints are most commonly adopted in clinical trials. The outcome measures from all the included SRs are mainly blood pressure and TCM symptom. It is probably related to the feasibility and operability either in inpatients or outpatients in small sample size and short-term clinical trials. Although it is helpful to reduce future cardiovascular risk to some extent by decreasing blood pressure and improving TCM symptoms, primary endpoints are widely recognized as more persuasive outcome measures when evaluating the efficacy of CHM for hypertension. Moreover, adverse effect, which is also very important in evaluating the safety of CHM, should be taken as outcome measures too. All of these problems affect the generation of high-level evidence of CHM for hypertension.

Ever since 1999 when the first Cochrane review of CHM was published [50], there is an increasing number of similar systematic reviews/meta-analysis. Thus, it is necessary to systematically identify and assess the quality of these reviews. The methodology and reporting quality of systematic reviews/meta-analyses of CHM have attracted great attention [51–54]. According to PRISMA statement, the quality of the current included SRs is judged as generally poor, especially those published in Chinese journals. Reviews had methodological and reporting flaws that could have influenced the reviews validity. The deficiencies mainly lies in searching literature, reporting of characteristics of included and excluded studies, extracting relevant data, evaluating primary trials' quality, and merging data. Also, the report of less persuasive outcome measures in most of the SRs has reduced the validity of the conclusions. So, in future, reviewers should attach more importance to the method of performing SR and receive relevant training of skills in reporting to reduce the amount of bias in their reviews. Researchers of clinical trials in TCM should also pay more attention to experimental design and methodological quality and improve the reporting quality according to the Consolidated Standards of Reporting Trials (CONSORT) statement [55], so as to improve the quality of TCM clinical research and ensure truth and reliability of conclusions. Although CHM appeared to be effective for hypertension in clinical use, most SRs were inconclusive that CHM had a definite effect for hypertension due to the poor evidence. 

More specifically, the following deficiencies in this overview should be taken into consideration before recommending the conclusion. Firstly, both the majority of included SRs and the original clinical trials are of low quality due to poorly designed and low-quality methodology. Secondly, as CHM is mainly used in China, SRs published in Chinese and English are retrieved. However, electronic databases in other languages have been omitted. Thirdly, unpublished studies and many negative randomized, double-blind, and controlled trials have not been taken into account for further analysis.

In summary, although both primary and secondary endpoints were all used to evaluate the effectiveness of CHM for hypertension, primary endpoints have not widely been used currently. Although this overview may show potential effectiveness of CHM for hypertension in terms of some outcome measures, most SRs failed to draw a confirmative conclusion for recommendation on the beneficial effect of CHM in hypertensive patients due to poor evidence. The benefits of CHM for hypertension still need to be confirmed in the future with more rigorous RCTs of more persuasive primary endpoints and high-quality SRs.

## Figures and Tables

**Table 1:** Outcome measures of CHM for hypertension in systematic reviews.

Outcome measures (number of SR)	Condition (number of SR)	CHM	First author	Number of RCTs/total	Conclusion	Risk of publication bias
Primary endpoints						

Mortality, stroke, coronary heart disease, and hypertensive renal damage (1)	Essential hypertension (1)	*Niuhuang Jiangya* preparation	Wang et al. (2008) [27]	3/3	B	NA
hypertensive renal damage (1)	Elderly hypertension (1)	Herbal products	Han (2012) [36]	4/45	A	NA

Secondary endpoints						

Blood pressure (10)	Essential hypertension (8)	*Niuhuang Jiangya* preparation	Wang (2008) [27]	3/3	B	NA
		Herbal products	Hu (2009) [28]	24/24	A	L
		Herbal products	Ren (2006) [29]	11/11	A	H
		*Tianma Gouteng* Yin	Dong (2011) [30]	6/6	A	L
		*Tianma Gouteng* Yin	Zhang (2012) [31]	0/0	B	NA
		Pinggan qianyang	Xu (2012) [32]	8/8	A	H
		Buyi shenqi	Shi (2012) [33]	5/5	A	H
		Buyi pishen	Liu (2011) [34]	13/15	A	L
	Elderly isolated systolic hypertension (1)	Herbal products	Li (2012) [35]	17/17	A	L
	Elderly hypertension (1)	Herbal products	Han (2012) [36]	45/45	A	NA
Triglycerides (1)	Essential hypertension (1)	Herbal products	Hu (2009) [28]	4/24	A	L
Pulse pressure (1)	Elderly isolated systolic hypertension (1)	Herbal products	Li (2012) [35]	4/17	A	L
Quality of life (1)	Elderly hypertension (1)	Herbal products	Han (2012) [36]	4/45	A	NA
TCM syndrome (5)	Essential hypertension (3)	Pinggan qianyang	Xu (2012) [32]	3/8	A	H
		Buyi shenqi	Shi (2012) [33]	4/5	A	H
		Buyi pishen	Liu (2011) [34]	9/15	A	L
	Elderly isolated systolic hypertension (1)	Herbal products	Li (2012) [35]	6/17	A	L
	Elderly hypertension (1)	Herbal products	Han (2012) [36]	4/45	A	NA

Notes: Pinggan qianyang: calming the liver and suppressing liver-yang to patients with hyperactivity of liver yang syndrome; Buyi shenqi: replenishing kidney qi to patients with kidney qi deficiency syndrome; Buyi pishen: replenishing spleen and kidney to patients with spleen and kidney deficiency syndrome; A: CHM may be or appear to be effective; B: the evidence is insufficient and inclusive; H: high; L: low; NA: not mentioned.
